# Molecular Genetic Analysis with Microsatellite-like Loci Reveals Specific Dairy-Associated and Environmental Populations of the Yeast *Geotrichum candidum*

**DOI:** 10.3390/microorganisms10010103

**Published:** 2022-01-04

**Authors:** Colin R. Tinsley, Noémie Jacques, Marine Lucas, Cécile Grondin, Jean-Luc Legras, Serge Casaregola

**Affiliations:** 1 Micalis Institute, INRAE, AgroParisTech, Université Paris-Saclay, 78350 Jouy-en-Josas, France; colin.tinlsey@agroparistech.fr (C.R.T.); Noemie.Jacques@inrae.fr (N.J.); Marine.Lucas@inrae.fr (M.L.); Cecile.Grondin@inrae.fr (C.G.); Serge.Casaregola@inrae.fr (S.C.); 2 Unité Microbiologie et Génétique Moléculaire, Department des Sciences de la Vie et Santé, AgroParisTech, 16 Rue Claude Bernard, 75005 Paris, France; 3 SPO, Université de Montpellier, INRAE, Institut Agro, 34000 Montpellier, France

**Keywords:** *Geotrichum* *candidum*, adaptation, environment, dairy

## Abstract

*Geotrichum candidum* is an environmental yeast, also found as part of the cheese surface microbiota, where it is important in the ripening of many traditional cheeses, such as Camembert. We have previously developed a Multi Locus Sequence Typing (MLST) scheme, which differentiated five clades, of which one contained only environmental isolates, two were composed almost entirely of dairy isolates, and two others contained a mixture of dairy, environmental, and miscellaneous food isolates. In order to provide a simple method to uniquely type *G. candidum* strains, and in addition to permit investigation of the population structure at a fine level, we describe here a molecular analysis using a set of twelve highly discriminating microsatellite-like markers. The present study consolidates the previously suggested division between dairy and environmental strains, and in addition distinguishes a specifically European group of environmental strains. This analysis permitted the discrimination of 72 genotypes from the collection of 80 isolates, while retaining the underlying meaningful phylogenetic relation between groups of strains.

## 1. Introduction

The dimorphic yeast *Geotrichum candidum* (teleomorph *Galactomyces candidus*) is an environmental species commonly found in foodstuffs, where it is present either as part of the technological microbiota, as in cheeses, or as a spoilage microorganism, and in addition is of interest as a source of enzymes (lipases, etc.) for biotechnological applications (reviewed in [1,2,3]). Owing to its propensity for filamentous growth, it was long considered as a fungus, as shown by the many names applied to this species since its description in 1809 by Link [4]. Its phylogenetic position has only recently been stabilized. Comparative genomic analysis has unambiguously assigned *G. candidum* to the Saccharomycotina subphylum [5]. In this study, genome analysis showed that *G. candidum* has specifically retained over 250 genes during evolution, some of which may partly explain its filamentous phenotype. In addition, this genome sequence analysis revealed a number of interesting genes which may be useful for biotechnology [6].

Due to the importance of *G. candidum* in the agrofood- and bio-industries, the population structure of this species has been the subject of considerable research. Based on the study of 62 isolates, it was shown that *G. candidum* displays significant polymorphism in its rDNA sequence, and 32 different sequences could be defined [7]. Intra-specific diversity was further studied in an MLST analysis involving 40 isolates, which defined distinct populations, some being specifically associated with cheese-making. In this publication, a correlation was found between two of these populations and categories of rDNA sequences [8], suggesting that rDNA sequence comparison may be used for structuring populations in this species. A more recent MLST analysis involving 67 isolates also defined distinct populations [9], one of these being composed exclusively of environmental strains. In addition, this analysis distinguished two types of dairy strains, one forming a homogeneous group with little genetic diversity, and the other more closely related to environmental isolates. Overall, this ensemble of results provides evidence suggesting an adaptation of *G. candidum* to the cheese-making environment.

Microsatellite analysis has proven to be a method of choice for molecular typing of yeast. The work of Legras and colleagues [10] established distinct groups of *Saccharomyces cerevisiae* directly linked to their different uses in biotechnology, which more recently has been supported by complete genome sequencing [11,12]. Since the mid-2000s, a number of studies have used this technique on food yeasts to study interspecies diversity [13], to distinguish populations [14,15,16,17], to show correlation between phenotypic and genotypic characteristics, or to bring to light correlations between geographic and genotypic characteristics [18,19,20,21].

Here, we selected a set of polymorphic trinucleotide motif-rich regions and apply a typing scheme using these microsatellite-like markers to the collection of isolates previously analysed by MLST and inter-LTR PCR typing [9], and show that we can clearly distinguish strains associated with cheese from those isolated in natural environments. Overall, our results strongly support a model whereby certain groups of *G. candidum* strains have been domesticated in cheese production and have since evolved separately from naturally occurring isolates.

## 2. Materials and Methods

### 2.1. Yeast Isolates and Growth Conditions

Isolates used in this study are listed in Table 1 and have been isolated in former projects. Thirty-nine of the 80 isolates (isolates prefixed “CNRZ” or “CLIB”) were from the CIRM-Levures Biological Resource Center, INRAe Montpellier, France; https://cirm-levures.bio-aware.com/ (accessed on 3 March 2020)). Twenty “FM” isolates were provided as part of the “Food Microbiomes” project (*Agence National de la Recherche*, France; ANR-08-ALIA-0007) by various industrial enterprises; information is available concerning the origins of 13 of these isolates, seven were anonymized in accordance with certain enterprises’ confidentiality policies. Other isolates were from the Westerdijk Institute (Utrecht, The Netherlands), the University of Caen (France), the Museum National d’Histoire Naturelle (Paris, France), VTT Technical Research Center (Finland), Kasetsart University (Bangkok, Thailand), the DSMZ-(Braunschweig, Germany), the BCCM/MUCL (Louvain la Neuve, Belgium), the NCYC (Norwich, United Kingdom), or the NITE (Chiba, Japan). Yeast strains were grown in YPD medium (Yeast Peptone Dextrose: yeast extract 10 g L^−1^, bacto peptone 10 g L^−1^, glucose 10 g L^−1^) at 28 °C with shaking. For solid medium, agar was added to YPD medium at 1.4% *w/v*.

### 2.2. DNA Extraction

The procedure used the “Nucleospin Plant II” kit (Macherey-Nagel, Hoerdt, France) with certain modifications, necessary for breaking down the resistant yeast cell walls. Cultures grown in 20 mL of YPD medium overnight at 28 °C were sedimented by centrifugation at 2500 *g* for 3 min. The cell pellets were washed in 5 mL of 10 mM EDTA, pH 8, and suspended in 3 mL of sorbitol buffer (1.2 M sorbitol, 100 mM EDTA-Na, 100 mM Tris-HCl, 35 mM beta-mercaptoethanol, pH 8) to which were added 50 units of zymolyase (Amsbio, Abingdon, UK). After incubation for 1 h at 37 °C, the cells were sedimented and the pellet suspended in 2 mL of lysis buffer according to kit manufacturer’s protocol. Digestion with 1 mg of proteinase K was carried out at 56 °C for 3 h, then the temperature was increased to 65 °C before addition of 250 µg of RNase A and incubation for a further 15 min. DNA purification was performed according to the manufacturer’s protocol, and the resulting genomic DNA was dissolved in 300 μL of TE buffer (10 mM EDTA-Na, 10 mM Tris-HCl, pH 8) containing 0.4 μg mL^−1^ RNase. The quality of the DNA preparation and its approximate concentration were estimated by agarose gel electrophoresis.

### 2.3. Loci Selection

In order to detect microsatellite, we searched for genomic enriched for specific trinucleotides using the program “Tandem Repeats Finder” v. 4.07b (TFR; Gary Benson, Boston University). While some of these loci were clear microsatellite loci, others did not present perfect microsatellite stretches, but were nonetheless seen to be polymorphic. Repeats between 100 and 400 bases in length in the index sequenced strain (CLIB 918) were selected, and those giving polymorphic amplicons between 100 and 500 bases in length in the tested strains were retained. Of the 13 loci identified by “Tandem Repeats Finder”, twelve were retained, the locus ML069 being of limited diversity with only 6 alleles in the populations studied (Table S1, Figure S1).

### 2.4. PCR Amplification

The primers used in this study were designed with Primer3 (http://fokker.wi.mit.edu/primer3, (accessed on 3 September 2014)) based on the sequences flanking microsatellite-like loci determined in the sequence of *G. candidum* [5] (Table S1). Multiplex PCRs were performed with primer mixes: (B3/B4, B5/B6, D1/D2, E5/E6, G7/G8), (A3/A4, A7/A8, D3/D4, F7/F8), and (A5/A6, I3/I4, I5/I6, F1/F2). Amplification used the enzyme Ex-Taq (TaKaRa) under the manufacturer’s recommended conditions with a cycle of 94 °C for 4 min, followed by 30 cycles of 94 °C for 30 s, 60 °C for 30 s, and 72 °C for 40 s. The program was terminated by 5 min at 72 °C. Fragment lengths were measured with a Beckman Coulter CEQ 8800 Genetic Analysis System.

### 2.5. Data Treatment

Measured amplicon lengths were converted to integer numbers of bases by an in-house clustering algorithm (Tables S2 and S3), then phylogenetic analysis was performed using the model of Edwards and Cavalli-Sforza [22,23,24] as implemented in the package “PoppR” in “R” [24,25]. It was not possible to distinguish haploid from diploid states in cases where a single band was produced from a given primer pair. Hence, data from the strains which only produced a single band per primer pair were converted to a “diploid” state by creating a second allele identical to the first.

As an alternative approach, one thousand “haploid” genotypes were created for each isolate by choosing with equal probability one of the two alleles at each locus for heterozygous loci or taking the unique allele in the case of haploid (homozygous) loci. One thousand neighbor-joining (NJ) trees were produced (1000 bootstraps per tree) and an average consensus tree was created by the phylogenetic program suite, “Mega X” [26].

Detection of the population structure was performed with “Instruct” [27]. The outputs of 27 runs were combined, and the best partitioning was determined as proposed by the program “CLUMPP” [28].

A comparison of results of the present study with those from MLST analysis [9] was performed with the Mantel test [29] implemented in “R” and by the online algorithm “Icong” (http://max2.ese.u-psud.fr/icong/index.help.html, (accessed on 10 March 2021)) [30]. The Pearson correlation between the two distance matrices was calculated in R, using the function “cor”.

## 3. Results and Discussion

The 80 isolates analyzed include the 64 previously studied by MLST analysis [9], with the addition of 16 further isolates specific to this study. The panel comprised 47 isolates from a dairy or cheese-making environment (the isolates from human stools were considered to be in this category), 18 environmental isolates from domestic, agricultural, or natural contexts, and 14 of unknown origin (Table 1).

The genome of strain *G. candidum* CLIB 918 (=ATCC 204307 [5]) was used to search for microsatellite loci. As markers, we chose twelve microsatellite-like regions rich in trinucleotide repeats giving amplicons between 100 and 500 bases in length, hence suitable for phylogenetic analysis (Table S1). Amplicon lengths corresponding to each of the markers with each of the isolates are shown in Tables S2 and, after clustering of the data to groups separated by integer base lengths, S3. Three of the isolates, CBS 178.71, CBS 615.84, and FM119 gave two PCR products with several (respectively 4, 4, and 7) of the primer pairs, and seven others gave two PCR products with one of the primer pairs, while the rest gave one amplicon (see Tables S2 and S3). Since it was not possible to discriminate the haploid from diploid homozygous states on the basis of these data, we treated all isolates as diploids.

**Figure 1:**
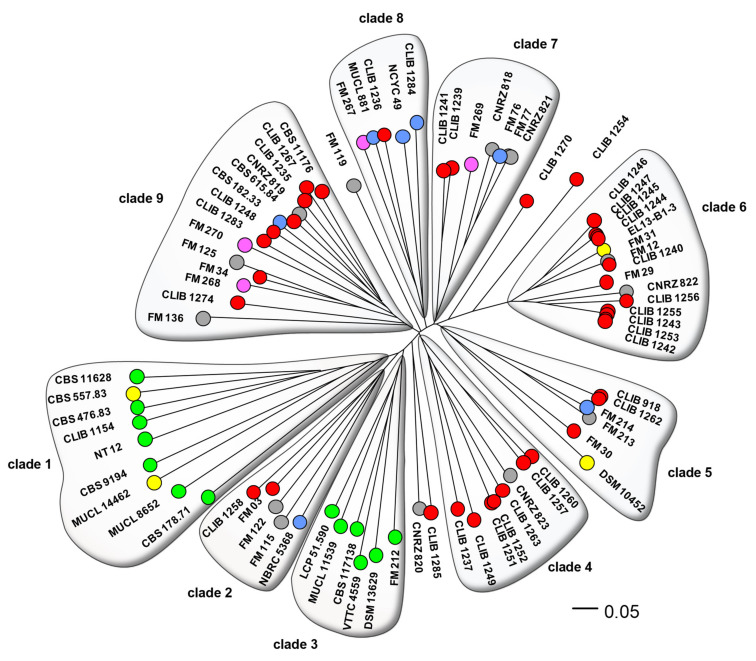
Dendrogram of the *G. candidum* strains. Phylogeny of the 80 isolates was calculated by the neighbor-joining algorithm using distances calculated according to Edwards and Cavalli-Sforza [22]. Genotypes are represented by coloured circles, according to the isolates’ origins: red, cheese; blue, milk; pink, human stools; yellow, foodstuffs; green, environmental; grey, origin unknown.

Figure 1 shows the neighbor-joining (NJ) tree resulting from analysis of the chosen microsatellite markers. This analysis discriminated 72 genotypes from the set of 80 isolates. The most important cluster of identical genotypes contained strains CLIB 1242, CLIB 1243, CLIB1253, and CLIB 1255 that were isolated on “Reblochon” cheese from two different factories separated by 15 km. The second cluster contained strains CLIB 1244, CLIB 1245, and CLIB 1247 isolated on “Reblochon” and “Tome de Savoie” cheeses in another factory, at a 15-km distance from the former two. A third pair of identical genotypes, CLIB 1251 and CLIB 1252, were isolated from “Epoisses” cheese in the same cheese factory. In contrast, CLIB 918 and CLIB 1262 had been isolated from “Pont l’Evêque” and “Saint Nectaire” cheeses in different regions of France, and the anonymization of the former of the FM12, CLIB 1240 pair prevents us from investigating their respective localizations. We note that most strains were not closely related, which suggests that they did not derive from few starter cultures. Indeed, we can expect that the use of starter cultures would lead to the presence of many groups of identical or very closely-related strains in the phylogram of Figure 1, in opposition to the diversity that is observed. This is in accordance with the period of isolation of CLIB and CNRZ strains, 20 to 30 years ago, when commercial ripening starters were not used for cheese making.

Overall, there is a clear distinction between environmental and cheese or other dairy isolates. Clades 1 and 3 contained exclusively environmental isolates (OTUs coloured green in Figure 1) or those from non-dairy foodstuff (OTUs coloured yellow). The two “food” isolates within one of these clades, CBS 557.83 and MUCL 14462, were respectively from peach and from squash, and can reasonably be assimilated to environmental isolates, since fruit is one of the natural habitats of *G. candidum* [32]. Clade 3 contained environmental strains exclusively of european origin, compared to the mostly non-european origins of the strains in clade 1. The other clades contained a mixture of cheese, human stool (very probably originating from dietary cheese), and dairy strains, with the exception of two isolates from food. Strains DSM 10452 was isolated from sauerkraut [33], while EL-13-B1-3 (origin given as ‘refrigerator’) may have been a contaminant originating from cheese (Figure 1). Isolates of unknown origin (CNRZ 818 to 823 and seven anonymized *G. candidum* of the “FM” group) were distributed throughout the phylogram, excepting the environmental clades. As these CNRZ strains are from a historic INRAE culture collection dedicated to dairy product microorganisms, it is logical to see them clustered with those from other dairy products. A similar situation can be seen for the anonymized isolates of the “Food microbiome” project that are also associated with dairy clades. There was no noticeable generalized clustering of strains according to cheese type or place of origin.

The five isolated strains (CLIB 1254, CLIB 1270, CLIB 1285, CNRZ 820, and FM 119) outside of the defined clades may represent poorly sampled clades. They might otherwise contain genetic material from two different sources, intermediate strains as seen in *Saccharomyces cerevisiae* [11,34]. Investigation showed that, excepting strain CLIB 1254 which harbored rare alleles at four of the twelve loci, these strains indeed contained mostly a mixture of alleles from the adjacent clades (Table S3).

It is interesting to compare the results of the present study with those of Morel, since the ploidy of about half of the strains has previously been inferred by analysis of the *MAT* locus. In the present work, three of the isolates (CBS 178.71, CBS 615.84, and FM 119) gave two PCR products for four or more of the 13 loci, and were considered very probably diploid. Another seven (CLIB 1235, CLIB 1249, CLIB 1254, EL13-B1-3, FM 213, FM 267, and MUCL 11539) gave two bands for only one of the loci, equivocal evidence for a diploid state since a local duplication would give the same result. Results for the analysis of the *MAT* loci are given in Table 1. Strains CBS 178.71, CLIB 1249, CLIB 1254, and CLIB 1284 were inferred to be diploid, since they carry both the *MATA* and *MATB* alleles, while another 36 are very probably haploid, being either *MATA* or *MATB*. Of the four afore-mentioned *MATA*/*MATB* strains, one (CLIB 1284) gave only one band for each of the 13 loci tested and the three others gave two bands for at least one microsatellite locus. These results were consistent with a diploid state, CLIB 1284 possibly being a result of mating between two closely related strains. Of the 36 strains possessing one only of *MATA* or *MATB*, two gave discordant results in this study: CLIB 1235 was apparently diploid for one locus, while CBS 615.84 gave two PCR products for four of the 13 loci and hence was very likely a diploid strain. The result for CLIB 1235 may be due to a partial duplication of the locus followed by mutation, or an introgression of DNA carrying the locus from another strain. The case of CBS 615.84 is more interesting, since it is the anamorphic type strain of the *G. candidum* species, perhaps indicating that the strain results from a mating followed by loss or erosion of one of the mating type loci.

Since we had a mixture of haploid and isolates strains, a second approach was used to consolidate the primary results. We calculated a consensus tree on the basis of 1000 rounds of random sampling of the alleles at each locus (Figure S2). This phylogram differed in some details from the tree shown in Figure 1, but produced the same clades as those previously defined and similarly separated environmental clades 1 and 3 from the other, cheese and dairy, clades with bootstrap values of 100 and 88%, respectively. The lower average values for bootstrap for the tree, compared to those observed with MLST [9], may result from the small number of loci (12 here) in comparison to the higher number of variable characters considered in an MLST study, as has been observed in other studies, which used less stringent resampling techniques [10,18]. Thus, these results support the robustness of both of the techniques for analysis of yeasts of mixed ploidy.

**Figure 2:**
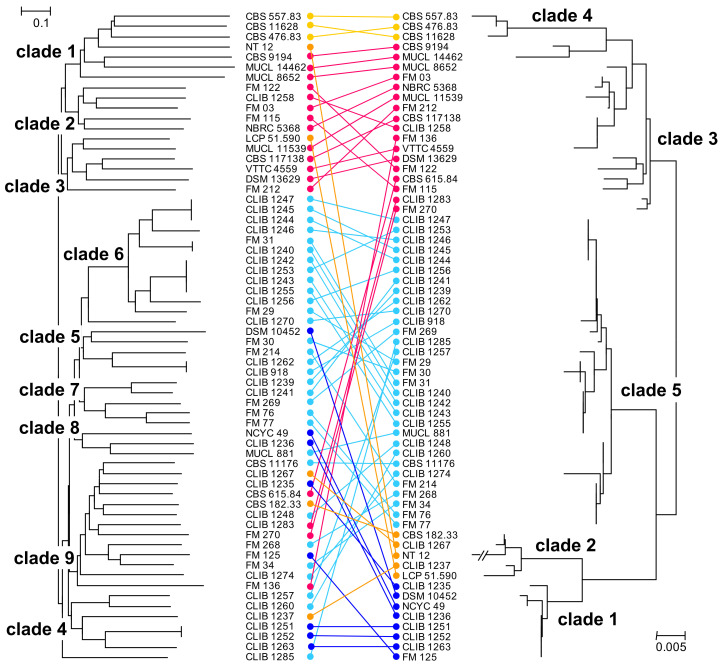
Comparison of NJ trees obtained by analysis of the microsatellite-like loci and MLST analyses of the *G. candidum* strains. Left, microsatellite analysis; right MLST analysis [9]. Taxon order was optimized manually using “Mega X” and strains that were not common to the two studies were removed. Connectors are coloured with respect to the MLST clades: dark blue, clade 1; orange, clade 2; pink, clade 3; yellow, clade 4; turquoise, clade 5.

We compared the results obtained with this set of microsatellite-like markers with those that we had obtained previously by MLST [9]. The Pearson correlation coefficient between the genetic distance matrices calculated from the MLST and from the microsatellite data was 0.58 and the similarity between the two distance matrices, as determined with the Mantel test, was significant at *p* = 0.0001. Another approach, comparing the topologies of the NJ trees (Icong [30]) gave a significance at *p* = 0.0007. The trees are represented face to face in Figure 2, demonstrating an overall similarity between the MLST and microsatellite phylogenies. In the previous work (phylogram on the right of the figure), two MLST clades, 1 and 5, contained the large majority of dairy isolates, while clades 3 and 4 corresponded mostly to environmental isolates. Isolates of the MLST clades 3, 4 (environmental), and 5 (dairy) were distinguished by the present microsatellite analysis, though the MLST dairy clades 1 and 2 were not defined here but rather redistributed within the phylogeny. Indeed, there is a good correlation between the MLST clades 3 and 4 and the environmental clades, 1 and 3, of the present study (Figure 2), and the two environmental strains of the MLST clade 2 (LCP 51.590 and NT12) are grouped by the present study together with the other environmental isolates. In addition, three dairy strains (FM270, CLIB 1283, and CBS 615.84) from MLST environmental clade 3 were more coherently placed by the present microsatellite analysis in clades together with other dairy isolates.

**Figure 3:**
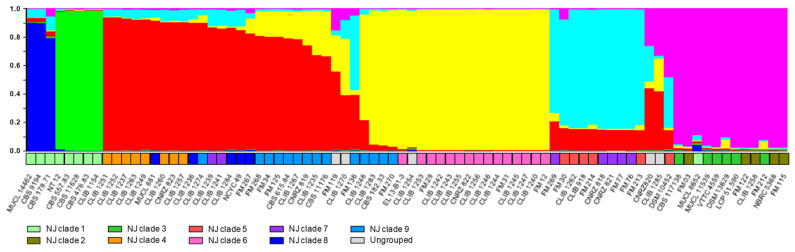
Analysis of microsatellite data and clustering of the *G. candidum* strains by “Instruct”. Colours of the histogram columns represent the proportion of the genetic constitution of an individual derived from each of the six hypothetical ancestral populations. For comparison with the analysis presented in Figure 1, the panel immediately above the isolates’ names shows the clades defined previously: light green, clade 1; khaki, clade 2; green, clade 3; orange, clade 4; red, clade 5; pink, clade 6; purple, clade 7; dark blue, clade 8; turquoise, clade 9; grey, strains not associated with named clades in the previous figure.

The extent of sexual reproduction is not known in this species, although the reticulated tree obtained from a previous MLST analysis revealed a number of genetic exchanges between the strains analyzed [9,31]. To account for genetic exchanges, we analysed the data using the program “Instruct” [27]. Based on the “Structure” algorithm [35], but not making the assumption of free mating and allowing populations to be inbred, the algorithm calculates the proportions of the genetic composition of an individual, which is derived from each of a number of hypothetical ancestral populations, the constitution and number, *K*, of these ancestral populations was estimated to maximize the prior probability of the model. Testing various values of *K* gave a cutoff value of 6, above which no significant increase in probability was seen. We conclude that the data are best explained by the existence of six ancestral populations. Results are shown in Figure 3, which also presents a comparison with the results from the NJ distance tree. The grouping of the isolates was similar in the two analyses. The environmental NJ clade 1 was separated from the others in the “Instruct” analysis, and furthermore split into two subgroups (groups predominantly deep blue and green in the upper part of Figure 3). However, neighbor-joining clades 2 and 3 were grouped together (“Instruct” cluster indicated in pink), leading to a less complete separation of environmental and dairy isolates.

A distinction between dairy and environmental strains is in agreement with the results of previous studies using RAPD [36] or MLST [8,37]. In our study, involving a large number of environmental isolates, it is interesting that the methods described all divided these isolates into the same two clades. This phylogenetic distance may represent geographical separation, since isolates belonging to clade 2 are all European, whereas those from clade 1, were mainly isolated in non-European countries (Africa, the Americas, and south-east Asia). Indeed, Table S4 shows that the former group contains a large majority of rare alleles at each of the loci, underlining their genetic distance from the other strains, including those environmental strains isolated in Europe. In support of the geographical explanation are the relatively large distances between strains of the non-European group, though no consistent subdivision on geographical bases is evident. The distance between the environmental and dairy clades seen in Figure 1 is confirmed in the analysis by “Instruct”, where environmental strain genotypes are mostly derived from two ancestral populations not found in the dairy isolates. Interestingly, the majority of French dairy isolates are distant from the environmental clades in the two analyses, suggesting a considerable lapse of time between domestication and the present-day cheese-making strains, with little genetic transfer (or transfer of strains) between the domesticated and environmental gene pools, or otherwise domestication from an environmental source geographically distant from France.

MLST or microsatellite typing methods have been used in many studies in the past. However, if discrimination between populations was often successful, correlations between the genotyping and the origin or the technological capabilities of the species studied were more difficult to detect. Microsatellite typing has proved very successful with the model species *S. cerevisiae,* since correlations between the technological activities and/or the geographic origin were observed [10] and later confirmed and extended by several genomic studies [11,12,14,34,38,39,40].

An ideal typing system would allow discrimination of each isolate while allowing the tracing of the underlying ancestry linking groups of strains. In practice phylogenetic trees are a compromise between these two, often contradictory objectives. The present microsatellite analysis clearly shows meaningful phylogenetic relationships, clustering individuals into coherent groups in relation to use, ecological, and geographical parameters. At the same time, we were able to define 72 genotypes in the collection of 80 isolates, probably a minimal estimate of the discriminatory power of the technique, since some of these, for example CLIB 1251 and CLIB 1252, may well represent the same organism isolated at different times from the same cheese cellar or from nearby locations.

As mentioned above, we detected no general differentiation between geographic origins within dairy yeasts or between the type of technology used in cheese-making, whereas closely related group of oenological strains have been well separated between flor and non-flor populations using microsatellites [14], a separation also confirmed by genomic analysis [39]. A similar situation to that of *G. candidum* was found for *Starmerella bacillaris* or for *Torulaspora delbrueckii* [41] whose oenological strains could be readily differentiated from natural isolates, but for which no specific distinction was made between different vineyards or wineries [20]. In this context, it will be of interest to apply global genomic analysis to *G. candidum*.

There are several ways in which yeast can adapt to a specific environment: selection for specific positive mutations or rearrangements [42], acquisition of genes through HGT [43,44,45], or specific retention of genes during evolution [5]. In the last of these studies, it was shown that *G. candidum* CLIB 918, isolated from St Nectaire cheese, presented 265 specifically retained ancestral genes and 16 genes acquired by HGT, though these genes which distinguished *G. candidum* from related species were not obviously related to functions or metabolic pathways associated with cheese making. The result of a population genomic study in *Geotrichum candidum* could shed light on the adaptive mechanism that permitted this yeast species to colonize cheese surface, and help to decipher the role of genes transferred horizontally.

## Figures and Tables

**Table 1:** Strains used in this study.

Isolate Name	Substrate of Isolation	Geographical Origin	Mating Type ^a^	MLST Sequence Type ^b^	MLST Clonal Complex ^b^
CBS 178.71	Soil polluted with oil	Germany	MATA/MATB	nd	nd
CBS 182.33	Yoghurt	Italy	MATB	6	2
CBS 476.83	Soil	Senegal	nd	34	4
CBS 557.83	Peach	Egypt	nd	35	4
CBS 615.84	Brie cheese	France	MATB	20	3
CBS 9194	Fruitfly	Brazil	MATA	8	3
CBS 11176	Bryndza cheese	Slovak republic, Žilina	MATB	19	5
CBS 11628	Soil	South Africa, Western Cape	nd	33	4
CBS 117138	Compost	Italy	nd	14	3
CLIB 918	Pont-l’Évêque cheese	France, Calvados	MATA	28	5
CLIB 1154	Flower	France, French Guiana	nd	nd	nd
CLIB 1235	Camembert cheese	France, Orne	MATA	5	1
CLIB 1236	Goat’s cheese	France, Manche	MATA	7	1
CLIB 1237	Cow milk	France, Orne	MATA	3	2
CLIB 1239	Mont d’Or cheese	France, Doubs	MATA	29	5
CLIB 1240	Reblochon cheese	France, Haute-Savoie	MATB	22	5
CLIB 1241	Mont d’Or cheese	France, Doubs	MATA	29	5
CLIB 1242	Reblochon cheese	France, Haute-Savoie	MATA	22	5
CLIB 1243	Reblochon cheese	France, Haute-Savoie	MATA	22	5
CLIB 1244	Tomme de Savoie cheese	France, Haute-Savoie	MATB	30	5
CLIB 1245	Reblochon cheese	France, Haute-Savoie	MATA	30	5
CLIB 1246	Reblochon cheese	France, Haute-Savoie	MATB	30	5
CLIB 1247	Tomme de Savoie cheese	France, Haute-Savoie	MATB	30	5
CLIB 1248	Reblochon cheese	France, Haute-Savoie	MATA	19	5
CLIB 1249	Mont d’Or cheese	France, Doubs	MATA/MATB	nd	nd
CLIB 1251	Epoisses cheese	France, Côte-d’Or	MATA	7	1
CLIB 1252	Epoisses cheese	France, Côte-d’Or	MATA	7	1
CLIB 1253	Reblochon cheese	France, Haute-Savoie	MATB	30	5
CLIB 1254	Reblochon cheese	France, Haute-Savoie	MATA/MATB	nd	nd
CLIB 1255	Reblochon cheese	France, Haute-Savoie	MATA	22	5
CLIB 1256	Reblochon cheese	France, Haute-Savoie	MATA	29	5
CLIB 1257	Saint Nectaire cheese	France, Puy-de-Dôme	MATA	21	5
CLIB 1258	Saint Nectaire cheese	France, Puy-de-Dôme	MATB	14	3
CLIB 1260	Saint Nectaire cheese	France, Puy-de-Dôme	MATA	19	5
CLIB 1262	Saint Nectaire cheese	France, Puy-de-Dôme	MATA	29	5
CLIB 1263	Saint Nectaire cheese	France, Puy-de-Dôme	MATB	7	1
CLIB 1267	Chaource cheese	France, Aube	MATA	2	2
CLIB 1270	Saint Nectaire cheese	France, Puy-de-Dôme	MATA	29	5
CLIB 1274	Reblochon cheese	France, Haute-Savoie	MATB	16	5
CLIB 1283	Pont-l’Évêque cheese	France, Calvados	MATB	10	3
CLIB 1284	Raw cream	France, Calvados	MATA/MATB	nd	nd
CLIB 1285	Livarot cheese	France, Calvados	MATA	25	5
CNRZ 818	unknown	unknown	nd	nd	nd
CNRZ 819	unknown	unknown	nd	nd	nd
CNRZ 820	unknown	unknown	nd	nd	nd
CNRZ 821	unknown	unknown	nd	nd	nd
CNRZ 822	unknown	unknown	nd	nd	nd
CNRZ 823	unknown	unknown	nd	nd	nd
DSM 10452	Sauerkraut	Germany	nd	31	1
DSM 13629	Polyurethane	United Kingdom	nd	38	3
EL13-B1-3	Refrigerator	France	nd	nd	nd
FM 03	Cheese contaminant	unknown	MATB	12	3
FM 12	unknown	unknown	nd	nd	nd
FM 29	Cheese	Auvergne	MATB	23	5
FM 30	Cheese	Auvergne	MATB	24	5
FM 31	Cheese	Auvergne	MATB	23	5
FM 34	Goat’s cheese	Auvergne	MATA	18	5
FM 76	Raw milk	France, Normandie	MATB	18	5
FM 77	unknown	unknown	MATB	18	5
FM 115	unknown	unknown	MATB	26	3
FM 119	unknown	unknown	MATA/MATB	nd	nd
FM 122	unknown	unknown	MATA	9	3
FM 125	unknown	unknown	MATA	7	1
FM 136	unknown	unknown	MATA	14	3
FM 212	Corn silage	France	MATB	13	3
FM 213	unknown	unknown	MATA/MATB	nd	nd
FM 214	Milk	France, Normandie	MATA	18	5
FM 267	Stools	France, Normandie	MATA/MATB	nd	nd
FM 268	Stools	France, Normandie	MATA	18	5
FM 269	Stools	France, Normandie	MATA	27	5
FM 270	Stools	France, Normandie	MATA	11	3
LCP 51.590	Sand	Spain, Burgos	MATA	4	2
MUCL 881	Milk	Belgium, Flemish Region	nd	22	5
MUCL 8652	Wet hay	Belgium, Flemish Region	nd	37	3
MUCL 11539	Polluted water	Great Britain	nd	32	3
MUCL 14462	Squash	USA, Pierce county	nd	39	3
NBRC 5368	Butter	Great Britain	nd	36	3
NCYC 49	Milk	Great Britain	nd	40	1
NT 12	Rain forest	Thailand	MATB	1	2
VTTC 4559	Malting	Sweden	MATB	15	3

nd: not determined; a: Mating type according to Morel [31]; b: MLST types and clonal complexes are according to Jacques and colleagues [9].

## Data Availability

Data is presented within this article and Supplementary Material.

## Supplementary Materials

The following are available online at https://www.mdpi.com/article/10.3390/microorganisms10010103/s1, Figure S1: Distribution of amplicon lengths for the chosen microsatellite-like loci in the tested strains. Figure S2: Phylogeny of the G. candidum strains, average of random haploid genotypes. Table S1: Microsatellites-like loci chosen for molecular typing: positions, properties and oligonucleotides used for amplification from the genomic DNA. Table S2: Lengths of microsatellite loci, raw data. Table S3, Corrected lengths of microsatellite loci. Table S4: Distribution of alleles within the strains studied.
