# Supplementary material for: Molecular Genetic Analysis with Microsatellite-like Loci Reveals Specific Dairy-Associated and Environmental Populations of the Yeast Geotrichum candidum
Source: Microorganisms. 2022 Jan 4;10(1):103. doi: 10.3390/microorganisms10010103 (PMC8780849; doi:10.3390/microorganisms10010103)
Supplement: Supplementary file 1 [file microorganisms-10-00103-s001.zip › Figure S1.pdf]

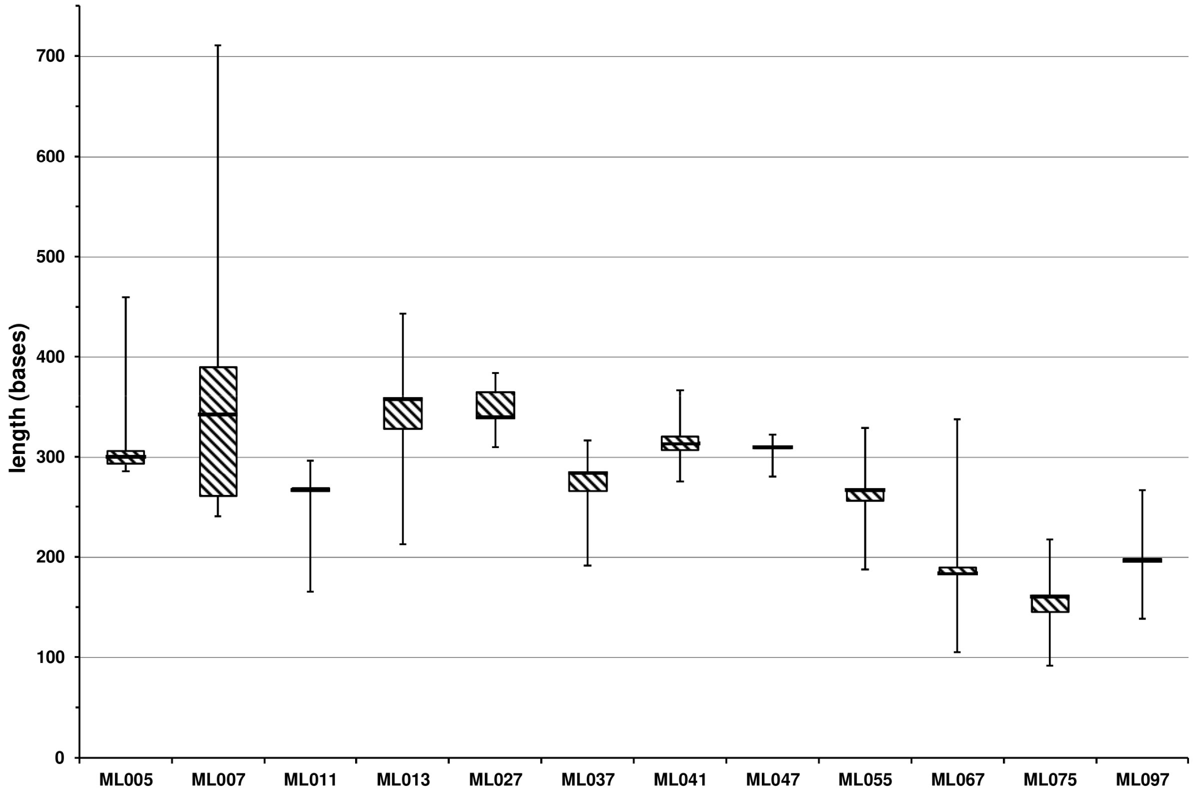

Figure S1. Distribution of amplicon lengths for the chosen microsatellite-like loci in the tested strains.
