# Supplementary material for: Molecular Genetic Analysis with Microsatellite-like Loci Reveals Specific Dairy-Associated and Environmental Populations of the Yeast Geotrichum candidum
Source: Microorganisms. 2022 Jan 4;10(1):103. doi: 10.3390/microorganisms10010103 (PMC8780849; doi:10.3390/microorganisms10010103)
Supplement: Supplementary file 1 [file microorganisms-10-00103-s001.zip › Table S1.pdf]

Table S1. Microsatellites-like loci chosen for molecular typing: positions, properties and oligonucleotides used for amplification from the genomic DNA.

Microsatellite name	Scaffold number	Start base of microsatellite sequence	End base of microsatellite sequence	Context	Repeat motif	Predicted amplicon length (repetitions) [a]	Oligonucleotide pair used for amplification	Sequences of oligonucleotides
ML005	3	1696696	1696985	CDS	GTT	290<br>(53)	A5<br>A6	GCAACACTGACTGACGCTTA<br>TCGTGACTCAGTTATGTCGGA
ML007	7	1166738	1167075	CDS	TGT	338<br>(81)	A7<br>A8	AGGATCCATTTGAATTGTACTTGGA<br>CCAGCCCCTAAACAATGCAAATA
ML011	16	358561	358830	CDS	CAA	270<br>(58)	B3<br>B4	ATCAGCGCTCGACACACT<br>AAGGCTGTGGTGGGGC
ML013	17	424091	424415	CDS	CAA	325<br>(79)	B5<br>B6	GCAGGGACAAGAACAATACCC<br>AGCAGCATGGAGTTGGCT
ML027	5	514292	514635	CDS	TTG	344<br>(97)	D3<br>D4	TCAAGGGCAAGCAACTTCAG<br>GCCATTGATAGAGCTTGCCC
ML037	12	666111	666397	CDS	CAA	287<br>(68)	E5<br>E6	CATCTCCGCTCGTACTCAGA<br>TGTGGCTGTGGTTGTTGAAG
ML041	20	64551	64869	CDS	CAG	319<br>(83)	F1<br>F2	CCGCCCCAGATGCTTCT<br>GCTGGAACTGTGGTCTTTGA
ML047	1	253966	254280	intergenic	AAG	315<br>(78)	F7<br>F8	TGGTTTATTGACGAACTAGACCG<br>CCCACTGGCTGCTTTTACTC
ML055	8	20524	20813	intergenic	ATA	290<br>(59)	G7<br>G8	AGGTGTGTAGTTCTAATGGCAATT<br>TCGAAATTAAAGATGGAAGCCCC
ML067	7	476425	476612	CDS	CAA	188<br>(44)	I3<br>I4	ACAGATGCAGATGTTGTACCAG<br>CTGACTGTTCCGGCTGAGA
ML069	15	234615	234771	CDS	TCA	157<br>(37)	I5<br>I6	TGGATGTATCTCTAGGCCGC<br>AACAACCAACAACAGCAGCA
ML075	4	1312567	1312717	intergenic	TTA	151<br>(32)	A3<br>A4	ACCGCTGCTGGTTTATTGTC<br>AAAAGTCGACACGGGAGACA
ML097	19	422927	423122	intergenic	AAT	196<br>(27)	D1<br>D2	CCATGGCTTTACTTGCACCA<br>GCCGAACCGATCAGCCATA

[a] Lengths in bases and number of repetitions present at the locus (in parentheses) are for amplicons from the type strain CLIB 918
