# Supplementary material for: Molecular Genetic Analysis with Microsatellite-like Loci Reveals Specific Dairy-Associated and Environmental Populations of the Yeast Geotrichum candidum
Source: Microorganisms. 2022 Jan 4;10(1):103. doi: 10.3390/microorganisms10010103 (PMC8780849; doi:10.3390/microorganisms10010103)
Supplement: Supplementary file 1 [file microorganisms-10-00103-s001.zip › Table S2.pdf]

Table S2. Lengths of microsatellite loci, raw data.

Strain	Length (measured) of amplicon produced at the microsatellite locus												
	ML005	ML007	ML011	ML013	ML027	ML037	ML041	ML047	ML055	ML067	ML069	ML075 *	ML097
CBS 11176	292.59	414.15	265.71	356.44	338.85	265.33	306.5	309.25	223.03	183.03	158.67	160.56	195.47
CBS 11628	421.96	393.37	291.89	386.16	350.88	259.16	318.00	289.61	252.46	206.71	158.55	-	262.62
CBS 117138	292.82	260.83	277.44	353.26	362.67	283.06	312.49	309.14	266.54	201.27	155.34	-	195.46
CBS 178.71	285.82	241.11/ 276.69	265.66	391.97	336.01/ 374.35	288.8	312.61	295.6/ 308.29	267.35	171.28/ 215.55	157.29	143.73	264.51
CBS 182.33	299.63	464.50	265.72	356.32	338.91	265.34	318.29	309.21	256.55	182.87	158.29	160.00	195.53
CBS 476.83	452.82	308.21	294.89	389.04	350.76	259.12	321.23	289.75	252.44	269.05	158.11	-	225.92
CBS 557.83	393.59	344.68	286.04	380.36	350.82	259.16	315.3	289.59	252.55	180.06	158.38	-	238.27
CBS 615.84	292.9/ 296.96	360.71/ 461.51	266.49	356.43	338.91/ 374.39	266.05	306.29	309.12	257.73	183.14/ 260.15	159.29	160.32	196.39
CBS 9194	301.75	282.00	279.62	327.83	359.81	303.41	274.59	280.24	328.12	336.82	158.45	144.66	205.52
CLIB 1154	459.10	452.83	294.96	388.69	351.22	287.13	332.45	289.93	252.58	212.86	157.22	-	259.39
CLIB 1235	312.05	338.62	266.17	356.42	338.99	265.75	306.94/ 365.41	309.35	258.3	182.84	152.22	160.40	19.006
CLIB 1236	299.58	342.02	265.62	356.37	374.38	265.26	312.31	309.25	256.43	182.96	158.53	163.68	195.32
CLIB 1237	296.45	366.81	265.69	359.33	374.33	283.05	306.75	309.4	257.54	189.34	158.47	144.75	195.28
CLIB 1239	292.78	363.86	265.60	353.59	338.88	282.96	306.69	309.27	267.40	182.99	158.54	144.65	195.31
CLIB 1240	302.70	260.80	265.87	356.42	338.97	286.17	318.26	309.20	266.81	182.92	158.37	160.42	195.69
CLIB 1241	292.71	363.80	265.61	353.53	338.93	282.95	306.80	309.20	266.42	182.92	158.39	145.00	195.33
CLIB 1242	302.71	261.00	265.59	356.37	338.98	265.23	318.19	309.34	266.33	182.96	158.39	160.44	195.31
CLIB 1243	302.68	261.25	265.60	356.36	339.06	265.24	318.01	309.27	266.35	183.06	158.56	160.33	195.31
CLIB 1244	305.86	260.88	265.61	356.30	339.00	285.88	318.22	309.34	266.39	183.02	158.49	160.00	195.36
CLIB 1245	305.85	261.04	265.61	356.38	339.08	285.90	318.21	309.38	266.36	182.99	158.44	160.34	195.3
CLIB 1246	305.87	260.95	265.95	355.43	339.03	285.22	318.22	309.42	266.91	182.84	158.38	160.39	195.68
CLIB 1247	305.95	261.27	265.55	356.38	339.09	285.83	318.1	309.33	266.27	182.95	158.49	160.43	195.3
CLIB 1248	299.64	382.77	265.85	356.5	339.00	265.45	306.84	309.41	259.81	182.92	158.42	160.46	195.6
CLIB 1249	317.47	347.05	266.21	353.93	374.78	284.05	307.50	309.55	257.46	188.07	157.14	160.16	185.18/ 195.12
CLIB 1251	321.65	341.9	265.99	353.64	374.45	265.58	306.78	309.35	256.96	189.28	158.46	163.51	195.73
CLIB 1252	322.11	342.1	265.58	353.52	374.42	265.22	306.56	309.45	256.32	189.08	158.66	163.00	195.33
CLIB 1253	302.75	260.75	265.98	356.53	338.86	265.56	318.20	309.21	266.94	182.91	158.42	160.00	195.64
CLIB 1254	302.02	259.99	266.37/ 286.72	356.58	338.92	285.79	318.05	309.22	267.71	183.53	158.74	159.32	196.14
CLIB 1255	302.74	261.08	265.57	356.35	339.01	265.21	318.19	309.36	266.28	182.96	158.45	160.52	195.31
CLIB 1256	302.66	261.14	286.15	356.46	339.08	265.84	318.1	309.43	266.75	183.1	158.54	160.45	195.61
CLIB 1257	292.50	691.00	265.57	318.55	338.93	282.9	306.53	309.21	256.32	189.33	158.59	154.18	195.31
CLIB 1258	295.96	261.00	265.57	370.41	362.81	282.89	321.42	308.43	265.17	189.24	155.31	141.73	195.33
CLIB 1260	292.47	710.00	266.06	318.69	338.93	283.41	306.59	309.26	258.07	189.46	158.62	154.08	195.86
CLIB 1262	296.57	348.38	265.59	318.58	339.03	282.94	312.09	309.19	288.16	182.87	158.59	151.18	195.53
CLIB 1263	292.55	341.67	265.58	353.48	374.32	282.83	306.43	309.38	288.19	189.40	158.63	163.44	195.36
CLIB 1267	292.55	388.08	266.07	356.22	338.94	265.65	306.41	308.42	258.18	182.99	158.76	160.22	195.96
CLIB 1270	293.36	344.88	265.60	356.51	338.36	282.99	312.38	309.22	266.43	182.93	158.49	160.00	195.28
CLIB 1274	308.94	301.39	266.45	356.83	338.94	266.00	306.74	309.36	257.59	189.22	158.32	163.41	196.31
CLIB 1283	302.93	323.75	265.61	356.36	339.01	265.26	306.62	309.02	266.42	236.32	158.56	160.59	195.33
CLIB 1284	311.26	337.7	265.95	318.68	374.00	283.52	306.90	308.28	256.8	182.92	157.41	159.37	195.66
CLIB 1285	296.32	503.5	266.49	356.54	362.63	283.85	318.21	309.07	257.64	189.19	158.35	160.43	196.39
CLIB 918	296.41	348.04	265.56	318.76	338.93	283.29	312.45	309.32	288.76	182.82	158.37	151.09	195.59

CNRZ 818	299.93	483.56	266.34	324.46	309.77	283.73	306.59	309.56	267.47	183.02	158.90	151.27	196.15
CNRZ 819	296.40	461.17	265.97	356.39	339.28	265.55	306.79	309.27	256.94	183.02	158.50	160.30	195.77
CNRZ 820	318.51	260.76	265.66	318.55	362.59	283.02	306.69	309.25	288.36	189.38	158.47	160.33	195.50
CNRZ 821	299.53	477.20	265.73	324.31	309.87	283.11	306.79	309.41	266.58	182.96	158.45	150.99	195.45
CNRZ 822	302.74	261.13	265.59	356.47	339.01	265.48	318.2	305.35	266.83	182.94	158.43	160.5	195.65
CNRZ 823	292.73	357.45	265.89	353.63	374.35	283.26	306.9	309.33	266.92	189.27	158.35	144.81	195.63
DSM 10452	292.59	422.82	265.57	312.91	383.07	282.94	312.25	315.96	256.29	182.92	158.49	92.2	195.32
DSM 13629	292.76	261.29	274.38	312.98	362.69	285.94	312.33	309.27	267.06	218.61	158.54	154.32	195.31
EL13-B1-3	301.58	260.85	265.72	356.41	339.20/ 374.66	286.23	318.6	309.54	266.56	182.9	157.24	160.26	195.43
FM 03	292.67	260.92	266.47	370.42	362.6	283.85	318.22	308.22	258.62	189.21	155.33	92.11	196.3
FM 115	298.45	477.05	265.84	350.43	365.54	283.14	312.30	308.28	265.53	206.78	158.54	92.12	195.55
FM 119	292.19/ 301.63	322.98	265.72	356.6/ 370.56	339.15/ 363.15	264.79/ 283.3	306.96/ 318.37	309.81	256.43/ 266.45	189.26/ 227.42	157.29	145.29	195.62
FM 12	302.72	261.08	265.59	356.35	339.08	285.88	318.11	309.43	266.33	182.98	158.47	160.43	195.29
FM 122	295.94	254.59	265.61	367.52	362.65	282.97	312.13	296.6	265.24	189.4	155.57	92.09	195.38
FM 125	292.84	388.06	266.4	359.41	338.77	265.96	306.59	309.16	257.58	183.08	159.05	160.42	192.29
FM 136	295.40	379.54	265.65	312.97	309.35	265.29	307.12	301.12	265.67	188.72	155.35	160.32	195.31
FM 212	289.92	260.91	265.59	350.43	362.71	282.94	318.3	309.33	267.08	206.64	155.2	104.52	195.4
FM 213	291.51	247.08/ 379.43	265.97	318.76	339.27	283.55	318.73	309.46	289.00	183.05	158.09	150.86	195.63
FM 214	292.49	379.69	265.65	318.12	339.07	283.00	317.99	309.39	288.23	183.04	158.45	151.02	195.3
FM 267	298.45	246.74/ 392.02	265.08	356.49	339.11	265.91	313.09	309.32	257.17	181.91	158.12	144.24	195.77
FM 268	292.46	391.5	265.62	359.2	339.06	265.26	306.99	309.35	256.42	185.93	158.47	160.48	195.31
FM 269	292.83	473.95	265.51	356.48	309.51	282.86	307.11	309.43	266.33	182.89	158.36	151.04	195.37
FM 270	305.81	535.37	265.63	356.34	338.97	265.27	308.84	309.38	256.45	236.25	158.41	160.39	195.34
FM 29	306.37	260.99	265.63	356.36	338.99	265.27	317.69	309.37	266.42	182.61	158.70	160.42	195.32
FM 30	299.64	529.78	265.59	312.88	339.04	282.94	312.25	309.24	265.26	182.91	158.43	144.88	195.46
FM 31	302.84	261.07	266.00	356.43	339.03	286.29	318.09	309.32	266.99	182.92	158.47	160.42	195.77
FM 34	292.59	388.18	265.83	359.47	339.03	265.44	306.82	309.38	256.75	182.99	158.45	160.43	195.61
FM 76	299.68	438.77	265.68	324.25	309.73	283.03	306.72	309.31	266.45	182.93	158.50	151.01	195.35
FM 77	299.41	477.38	265.81	324.23	309.52	283.14	306.65	309.32	266.68	183.12	158.67	151.10	195.48
LCP 51.590	293.20	261.07	265.59	370.46	362.74	282.95	312.39	309.39	277.25	224.49	155.31	92.19	195.27
MUCL 11539	322.71	261.28	265.64	307.23	362.63	282.97	312.24	309.28/ 320.84	277.23	215.62	158.75	144.99	195.42
MUCL 14462	304.88	292.16	270.81	442.09	359.92	315.17	274.55	290.82	275.91	289.83	158.55	216.17	205.78
MUCL 8652	292.87	260.92	165.67	212.75	365.45	191.77	317.37	308.59	188.39	105.09	156.3	92.10	138.51
MUCL 881	296.03	363.95	265.63	356.55	374.47	265.28	312.52	309.17	256.42	250.89	158.39	-	195.28
NBRC 5368	289.87	260.88	265.8	314.14	365.51	283.27	289.25	308.27	265.68	189.25	155.24	-	195.60
NCYC 49	312.02	662.00	265.63	318.56	374.36	265.27	312.20	309.33	266.39	183.00	158.39	144.75	195.37
NT 12	378.13	355.21	292.49	386.42	353.75	268.51	331.71	289.71	253.49	210.04	159.25	-	226.89
VTTC 4559	292.87	260.73	277.60	353.31	362.68	283.25	312.08	309.37	265.7	218.57	155.45	144.80	210.90

*) "-" : No PCR product was seen in a small proportion of cases. In this case the sequences flanking the locus ML075 are presumably less conserved than those of other loci.
