# Supplementary material for: Molecular Genetic Analysis with Microsatellite-like Loci Reveals Specific Dairy-Associated and Environmental Populations of the Yeast Geotrichum candidum
Source: Microorganisms. 2022 Jan 4;10(1):103. doi: 10.3390/microorganisms10010103 (PMC8780849; doi:10.3390/microorganisms10010103)
Supplement: Supplementary file 1 [file microorganisms-10-00103-s001.zip › Table S3.pdf]

**Table S3**. Corrected lengths of microsatellite loci.

| Strain | Length (bases) of amplicon produced at the microsatellite locus * | | | | | | | | | | | | |
|---|---|---|---|---|---|---|---|---|---|---|---|---|---|
| | ML005 | ML007 | ML011 | ML013 | ML027 | ML037 | ML041 | ML047 | ML055 | ML067 | ML069 | ML075 | ML097 |
| CBS 11176 | 293 | 414 | 266 | 356 | 339 | 265 | 307 | 309 | 223 | 183 | 158 | 160 | 196 |
| CBS 11628 | 422 | 392 | 292 | 386 | 351 | 259 | 318 | 290 | 253 | 207 | 158 | - | 263 |
| CBS 117138 | 293 | 261 | 278 | 354 | 363 | 283 | 312 | 309 | 266 | 201 | 158 | - | 196 |
| CBS 178.71 | 286 | 241, 277 | 266 | 392 | 336, 374 | 289 | 312 | 296, 309 | 266 | 171, 216 | 158 | 145 | 265 |
| CBS 182.33 | 299 | 464 | 266 | 356 | 339 | 265 | 318 | 309 | 257 | 183 | 158 | 160 | 196 |
| CBS 476.83 | 453 | 308 | 295 | 389 | 351 | 259 | 321 | 290 | 253 | 269 | 158 | - | 226 |
| CBS 557.83 | 394 | 345 | 286 | 380 | 351 | 259 | 315 | 290 | 253 | 180 | 158 | - | 238 |
| CBS 615.84 | 293, 296 | 361, 461 | 266 | 356 | 339, 374 | 265 | 307 | 309 | 257 | 183, 260 | 158 | 160 | 196 |
| CBS 9194 | 302 | 282 | 280 | 328 | 360 | 303 | 275 | 280 | 328 | 337 | 158 | 145 | 206 |
| CLIB 1154 | 459 | 453 | 295 | 389 | 351 | 286 | 332 | 290 | 253 | 213 | 158 | - | 259 |
| CLIB 1235 | 312 | 338 | 266 | 356 | 339 | 265 | 307, 365 | 309 | 257 | 183 | 152 | 160 | 196 |
| CLIB 1236 | 299 | 342 | 266 | 356 | 374 | 265 | 312 | 309 | 257 | 183 | 158 | 163 | 196 |
| CLIB 1237 | 296 | 367 | 266 | 359 | 374 | 283 | 307 | 309 | 257 | 189 | 158 | 145 | 196 |
| CLIB 1239 | 293 | 364 | 266 | 354 | 339 | 283 | 307 | 309 | 266 | 183 | 158 | 145 | 196 |
| CLIB 1240 | 302 | 261 | 266 | 356 | 339 | 286 | 318 | 309 | 266 | 183 | 158 | 160 | 196 |
| CLIB 1241 | 293 | 364 | 266 | 354 | 339 | 283 | 307 | 309 | 266 | 183 | 158 | 145 | 196 |
| CLIB 1242 | 302 | 261 | 266 | 356 | 339 | 265 | 318 | 309 | 266 | 183 | 158 | 160 | 196 |
| CLIB 1243 | 302 | 261 | 266 | 356 | 339 | 265 | 318 | 309 | 266 | 183 | 158 | 160 | 196 |
| CLIB 1244 | 306 | 261 | 266 | 356 | 339 | 286 | 318 | 309 | 266 | 183 | 158 | 160 | 196 |
| CLIB 1245 | 306 | 261 | 266 | 356 | 339 | 286 | 318 | 309 | 266 | 183 | 158 | 160 | 196 |
| CLIB 1246 | 306 | 261 | 266 | 356 | 339 | 286 | 318 | 309 | 266 | 183 | 158 | 160 | 196 |
| CLIB 1247 | 306 | 261 | 266 | 356 | 339 | 286 | 318 | 309 | 266 | 183 | 158 | 160 | 196 |
| CLIB 1248 | 299 | 383 | 266 | 356 | 339 | 265 | 307 | 309 | 260 | 183 | 158 | 160 | 196 |
| CLIB 1249 | 317 | 348 | 266 | 354 | 374 | 283 | 307 | 309 | 257 | 189 | 158 | 160 | 185, 196 |
| CLIB 1251 | 322 | 342 | 266 | 354 | 374 | 265 | 307 | 309 | 257 | 189 | 158 | 163 | 196 |
| CLIB 1252 | 322 | 342 | 266 | 354 | 374 | 265 | 307 | 309 | 257 | 189 | 158 | 163 | 196 |
| CLIB 1253 | 302 | 261 | 266 | 356 | 339 | 265 | 318 | 309 | 266 | 183 | 158 | 160 | 196 |
| CLIB 1254 | 302 | 261 | 266, 286 | 356 | 339 | 286 | 318 | 309 | 266 | 183 | 158 | 160 | 196 |
| CLIB 1255 | 302 | 261 | 266 | 356 | 339 | 265 | 318 | 309 | 266 | 183 | 158 | 160 | 196 |
| CLIB 1256 | 302 | 261 | 286 | 356 | 339 | 265 | 318 | 309 | 266 | 183 | 158 | 160 | 196 |
| CLIB 1257 | 293 | 691 | 266 | 319 | 339 | 283 | 307 | 309 | 257 | 189 | 158 | 154 | 196 |

| | | | | | | | | | | | | | |
|---|---|---|---|---|---|---|---|---|---|---|---|---|---|
| CLIB 1258 | 296 | 261 | 266 | 370 | 363 | 283 | 321 | 309 | 266 | 189 | 158 | 142 | 196 |
| CLIB 1260 | 293 | 710 | 266 | 319 | 339 | 283 | 307 | 309 | 257 | 189 | 158 | 154 | 196 |
| CLIB 1262 | 296 | 348 | 266 | 319 | 339 | 283 | 312 | 309 | 288 | 183 | 158 | 151 | 196 |
| CLIB 1263 | 293 | 342 | 266 | 354 | 374 | 283 | 307 | 309 | 288 | 189 | 158 | 163 | 196 |
| CLIB 1267 | 293 | 388 | 266 | 356 | 339 | 265 | 307 | 309 | 257 | 183 | 158 | 160 | 196 |
| CLIB 1270 | 293 | 345 | 266 | 356 | 339 | 283 | 312 | 309 | 266 | 183 | 158 | 160 | 196 |
| CLIB 1274 | 309 | 301 | 266 | 356 | 339 | 265 | 307 | 309 | 257 | 189 | 158 | 163 | 196 |
| CLIB 1283 | 302 | 323 | 266 | 356 | 339 | 265 | 307 | 309 | 266 | 236 | 158 | 160 | 196 |
| CLIB 1284 | 312 | 338 | 266 | 319 | 374 | 283 | 307 | 309 | 257 | 183 | 158 | 160 | 196 |
| CLIB 1285 | 296 | 504 | 266 | 356 | 363 | 283 | 318 | 309 | 257 | 189 | 158 | 160 | 196 |
| CLIB 918 | 296 | 348 | 266 | 319 | 339 | 283 | 312 | 309 | 288 | 183 | 158 | 151 | 196 |
| CNRZ 818 | 299 | 484 | 266 | 324 | 310 | 283 | 307 | 309 | 266 | 183 | 158 | 151 | 196 |
| CNRZ 819 | 296 | 461 | 266 | 356 | 339 | 265 | 307 | 309 | 257 | 183 | 158 | 160 | 196 |
| CNRZ 820 | 319 | 261 | 266 | 319 | 363 | 283 | 307 | 309 | 288 | 189 | 158 | 160 | 196 |
| CNRZ 821 | 299 | 477 | 266 | 324 | 310 | 283 | 307 | 309 | 266 | 183 | 158 | 151 | 196 |
| CNRZ 822 | 302 | 261 | 266 | 356 | 339 | 265 | 318 | 305 | 266 | 183 | 158 | 160 | 196 |
| CNRZ 823 | 293 | 357 | 266 | 354 | 374 | 283 | 307 | 309 | 266 | 189 | 158 | 145 | 196 |
| DSM 10452 | 293 | 423 | 266 | 313 | 383 | 283 | 312 | 316 | 257 | 183 | 158 | 92 | 196 |
| DSM 13629 | 293 | 261 | 274 | 313 | 363 | 286 | 312 | 309 | 266 | 219 | 158 | 154 | 196 |
| EL13-B1-3 | 302 | 261 | 266 | 356 | 339, 374 | 286 | 318 | 309 | 266 | 183 | 158 | 160 | 196 |
| FM 03 | 293 | 261 | 266 | 370 | 363 | 283 | 318 | 309 | 257 | 189 | 158 | 92 | 196 |
| FM 115 | 299 | 477 | 266 | 350 | 366 | 283 | 312 | 309 | 266 | 207 | 158 | 92 | 196 |
| FM 119 | 293, 302 | 323 | 266 | 356, 370 | 339, 363 | 265, 283 | 307, 318 | 309 | 257, 266 | 189, 227 | 158 | 145 | 196 |
| FM 12 | 302 | 261 | 266 | 356 | 339 | 286 | 318 | 309 | 266 | 183 | 158 | 160 | 196 |
| FM 122 | 296 | 255 | 266 | 368 | 363 | 283 | 312 | 296 | 266 | 189 | 158 | 92 | 196 |
| FM 125 | 293 | 388 | 266 | 359 | 339 | 265 | 307 | 309 | 257 | 183 | 158 | 160 | 192 |
| FM 136 | 296 | 380 | 266 | 313 | 310 | 265 | 307 | 301 | 266 | 189 | 158 | 160 | 196 |
| FM 212 | 290 | 261 | 266 | 350 | 363 | 283 | 318 | 309 | 266 | 207 | 158 | 105 | 196 |
| FM 213 | 293 | 247, 380 | 266 | 319 | 339 | 283 | 318 | 309 | 288 | 183 | 158 | 151 | 196 |
| FM 214 | 293 | 380 | 266 | 319 | 339 | 283 | 318 | 309 | 288 | 183 | 158 | 151 | 196 |
| FM 267 | 299 | 247, 392 | 266 | 356 | 339 | 265 | 312 | 309 | 257 | 183 | 158 | 145 | 196 |
| FM 268 | 293 | 392 | 266 | 359 | 339 | 265 | 307 | 309 | 257 | 186 | 158 | 160 | 196 |
| FM 269 | 293 | 474 | 266 | 356 | 310 | 283 | 307 | 309 | 266 | 183 | 158 | 151 | 196 |
| FM 270 | 306 | 535 | 266 | 356 | 339 | 265 | 309 | 309 | 257 | 236 | 158 | 160 | 196 |
| FM 29 | 306 | 261 | 266 | 356 | 339 | 265 | 318 | 309 | 266 | 183 | 158 | 160 | 196 |

| | | | | | | | | | | | | | |
|---|---|---|---|---|---|---|---|---|---|---|---|---|---|
| FM 30 | 299 | 530 | 266 | 313 | 339 | 283 | 312 | 309 | 266 | 183 | 158 | 145 | 196 |
| FM 31 | 302 | 261 | 266 | 356 | 339 | 286 | 318 | 309 | 266 | 183 | 158 | 160 | 196 |
| FM 34 | 293 | 388 | 266 | 359 | 339 | 265 | 307 | 309 | 257 | 183 | 158 | 160 | 196 |
| FM 76 | 299 | 439 | 266 | 324 | 310 | 283 | 307 | 309 | 266 | 183 | 158 | 151 | 196 |
| FM 77 | 299 | 477 | 266 | 324 | 310 | 283 | 307 | 309 | 266 | 183 | 158 | 151 | 196 |
| LCP 51.590 (CLIB 1415) | 293 | 261 | 266 | 370 | 363 | 283 | 312 | 309 | 277 | 224 | 158 | 92 | 196 |
| MUCL 11539 | 322 | 261 | 266 | 307 | 363 | 283 | 312 | 309, 321 | 277 | 216 | 158 | 145 | 196 |
| MUCL 14462 | 306 | 292 | 271 | 442 | 360 | 315 | 275 | 290 | 277 | 290 | 158 | 216 | 206 |
| MUCL 8652 | 293 | 261 | 166 | 213 | 366 | 192 | 318 | 309 | 188 | 105 | 158 | 92 | 139 |
| MUCL 881 | 296 | 364 | 266 | 356 | 374 | 265 | 312 | 309 | 257 | 251 | 158 | - | 196 |
| NBRC 5368 | 290 | 261 | 266 | 313 | 366 | 283 | 289 | 309 | 266 | 189 | 158 | - | 196 |
| NCYC 49 | 312 | 662 | 266 | 319 | 374 | 265 | 312 | 309 | 266 | 183 | 158 | 145 | 196 |
| NT 12 | 378 | 355 | 292 | 386 | 354 | 269 | 332 | 290 | 253 | 210 | 158 | - | 226 |
| VTTC 4559 | 293 | 261 | 278 | 354 | 363 | 283 | 312 | 309 | 266 | 219 | 158 | 145 | 211 |

*) Where two values are indicated for a given strain and locus, it is probable that the strain is diploid heterozygous at least for this locus. Where one value is given, no conclusion may be drawn concerning the ploidy of the strain.
