# Supplementary material for: Molecular Genetic Analysis with Microsatellite-like Loci Reveals Specific Dairy-Associated and Environmental Populations of the Yeast Geotrichum candidum
Source: Microorganisms. 2022 Jan 4;10(1):103. doi: 10.3390/microorganisms10010103 (PMC8780849; doi:10.3390/microorganisms10010103)
Supplement: Supplementary file 1 [file microorganisms-10-00103-s001.zip › Table S4.pdf]

Table S4. Strains used in this study.

	Presence of the strain's allele at a given locus in the other strains *											
Strain	ML005	ML007	ML011	ML013	ML027	ML037	ML041	ML047	ML055	ML067	ML075	ML097
CBS 11628	**0.0**	**0.0**	**0.1**	**0.1**	0.3	**0.2**	4.4	0.4	0.3	0.4	-	**0.0**
CBS 557.83	**0.0**	1.0	1.1	**0.0**	0.3	**0.2**	**0.0**	0.4	0.3	**0.0**	-	**0.0**
CBS 476.83	**0.0**	**0.0**	**0.1**	**0.1**	0.3	**0.2**	**0.2**	0.4	0.3	**0.0**	-	**0.0**
CLIB 1154	**0.0**	**0.0**	**0.1**	**0.1**	0.3	**0.0**	**0.1**	0.4	0.3	**0.0**	-	**0.0**
NT 12	**0.0**	**0.0**	**0.1**	**0.1**	**0.0**	**0.0**	**0.1**	0.4	**0.0**	**0.0**	-	**0.0**
CBS 9194	2.1	**0.0**	**0.0**	**0.0**	**0.1**	**0.0**	**0.1**	**0.0**	**0.0**	**0.0**	2.2	**0.1**
MUCL 14462	**0.0**	**0.0**	**0.0**	**0.0**	**0.1**	**0.0**	**0.1**	**0.0**	**0.0**	**0.0**	**0.0**	**0.1**
MUCL 8652	4.6	3.1	**0.0**	**0.0**	0.4	**0.0**	4.4	1.2	**0.0**	**0.0**	0.9	**0.0**
CBS 178.71	**0.0**	0, 0	11.4	**0.0**	0, 1.7	**0.0**	3.7	0, 1.2	1.3	0, 0.2	**0.2**	**0.0**
CLIB 1258	1.5	3.0	11.3	1.4	3.4	7.6	**0.1**	1.1	1.0	3.6	**0.0**	11.6
FM 03	4.5	3.0	11.3	1.4	3.4	7.6	4.3	1.1	1.2	3.6	0.8	11.6
FM 122	1.5	**0.0**	11.3	**0.0**	3.4	7.6	3.6	**0.0**	1.0	3.6	0.8	11.6
FM 115	**0.2**	0.3	11.3	**0.2**	0.3	7.6	3.6	1.1	1.0	0.3	0.8	11.6
NBRC 5368	**0.2**	3.0	11.3	**0.0**	0.3	7.6	**0.0**	1.1	1.0	3.6	-	11.6
LCP 51.590	4.5	3.0	11.4	1.4	3.4	7.6	3.6	10.3	**0.2**	**0.0**	0.9	11.6
MUCL 11539	**0.0**	3.0	11.4	**0.0**	3.4	7.6	3.6	10.3, **0.0**	**0.2**	**0.1**	2.2	11.6
CBS 117138	4.5	3.0	**0.2**	1.1	3.4	7.6	3.6	10.3	4.4	**0.0**	-	11.6
VTTC 4559	4.5	3.0	**0.2**	1.1	3.4	7.6	3.6	10.3	1.0	**0.2**	2.2	**0.0**

DSM 13629	4.5	3.0	**0.0**	0.4	3.4	1.5	3.6	10.3	4.4	**0.2**	**0.2**	11.6
FM 212	**0.2**	3.0	11.4	**0.2**	3.4	7.6	4.3	10.3	4.4	0.3	**0.0**	11.6
CNRZ 820	**0.0**	2.7	11.0	1.3	3.1	7.3	4.1	9.9	0.8	3.3	3.6	11.3
CLIB 1285	1.2	**0.0**	11.0	5.4	3.1	7.3	4.0	9.9	0.9	3.3	3.6	11.3
CLIB 1237	1.6	**0.0**	11.4	**0.2**	1.7	7.7	4.4	10.3	1.3	3.7	2.2	11.6
CLIB 1249	**0.0**	**0.0**	11.4	1.1	1.7	7.7	4.4	10.3	1.3	**0.0**	3.9	**0.0**, 11.6
CLIB 1251	**0.1**	0.4	11.4	1.1	1.7	3.4	4.4	10.3	2.6	3.7	0.5	11.6
CLIB 1252	**0.1**	0.4	11.4	1.1	1.7	3.4	4.4	10.3	2.6	3.7	0.5	11.6
CLIB 1263	4.5	0.4	11.4	1.1	1.7	7.7	4.4	10.3	1.2	3.7	0.5	11.6
CNRZ 823	4.5	**0.0**	11.4	1.1	1.7	7.7	4.4	10.3	4.5	3.7	2.2	11.6
CLIB 1257	4.5	**0.0**	11.4	1.7	6.4	7.7	4.4	10.3	2.6	3.7	0.3	11.6
CLIB 1260	4.5	**0.0**	11.4	1.7	6.4	7.7	4.4	10.3	1.3	3.7	0.3	11.6
DSM 10452	4.5	**0.0**	11.4	0.4	**0.0**	7.6	3.6	**0.0**	2.5	6.1	0.9	11.6
FM 30	0.9	**0.0**	11.4	0.4	6.3	7.6	3.6	10.3	1.0	6.1	2.2	11.6
FM 213	**0.0**	**0.2**, **0.2**	11.4	1.7	6.3	7.6	4.3	10.3	1.1	6.1	1.2	11.6
FM 214	4.5	**0.2**	11.4	1.7	6.3	7.6	4.3	10.3	1.1	6.1	1.2	11.6
CLIB 1262	1.5	**0.2**	11.4	1.7	6.3	7.6	3.6	10.3	1.1	6.1	1.2	11.6
CLIB 918	1.5	**0.2**	11.4	1.7	6.3	7.6	3.6	10.3	1.1	6.1	1.2	11.6
CLIB 1242	0.6	3.1	11.5	5.8	6.4	3.4	4.4	10.4	4.5	6.2	4.0	11.7
CLIB 1253	0.6	3.1	11.5	5.8	6.4	3.4	4.4	10.4	4.5	6.2	4.0	11.7

CLIB 1243	0.6	3.1	11.5	5.8	6.4	3.4	4.4	10.4	4.5	6.2	4.0	11.7
CLIB 1255	0.6	3.1	11.5	5.8	6.4	3.4	4.4	10.4	4.5	6.2	4.0	11.7
CLIB 1256	0.6	3.1	1.1	5.8	6.4	3.4	4.4	10.4	4.5	6.2	4.0	11.7
CNRZ 822	0.6	3.1	11.5	5.8	6.4	3.4	4.4	**0.0**	4.5	6.2	4.0	11.7
FM 29	0.3	3.1	11.5	5.8	6.4	3.4	4.4	10.4	4.5	6.2	4.0	11.7
CLIB 1240	0.6	3.1	11.5	5.8	6.4	1.6	4.4	10.4	4.5	6.2	4.0	11.7
FM 12	0.6	3.1	11.5	5.8	6.4	1.6	4.4	10.4	4.5	6.2	4.0	11.7
FM 31	0.6	3.1	11.5	5.8	6.4	1.6	4.4	10.4	4.5	6.2	4.0	11.7
EL13-B1-3	2.1	3.1	11.5	5.8	6.4, 1.7	1.6	4.4	10.4	4.5	6.2	4.0	11.7
CLIB 1244	0.3	3.1	11.5	5.8	6.4	1.6	4.4	10.4	4.5	6.2	4.0	11.7
CLIB 1245	0.3	3.1	11.5	5.8	6.4	1.6	4.4	10.4	4.5	6.2	4.0	11.7
CLIB 1247	0.3	3.1	11.5	5.8	6.4	1.6	4.4	10.4	4.5	6.2	4.0	11.7
CLIB 1246	0.3	3.1	11.5	**0.0**	6.4	1.6	4.4	10.4	4.5	6.2	4.0	11.7
CLIB 1254	1.2	**0.0**	10.5, **0.2**	4.9	5.5	0.7	3.5	9.4	0.4	5.2	**0.2**	10.8
CLIB 1270	3.7	**0.1**	10.5	4.9	5.5	6.8	2.8	9.4	3.6	5.2	3.1	10.8
CNRZ 821	0.9	0.3	11.4	0.4	0.6	7.6	4.4	10.3	4.5	6.1	1.2	11.6
FM 77	0.9	0.3	11.4	0.4	0.6	7.6	4.4	10.3	4.5	6.1	1.2	11.6
FM 76	0.9	**0.0**	11.4	0.4	0.6	7.6	4.4	10.3	4.5	6.1	1.2	11.6
CNRZ 818	0.9	**0.0**	11.4	0.4	0.6	7.6	4.4	10.3	1.3	6.1	1.2	11.6
FM 269	4.5	**0.0**	11.4	5.7	0.6	7.6	4.4	10.3	4.5	6.1	1.2	11.6

CLIB 1239	4.5	0.3	11.4	1.1	6.4	7.6	4.4	10.3	1.3	6.1	2.2	11.6
CLIB 1241	4.5	0.3	11.4	1.1	6.4	7.6	4.4	10.3	4.5	6.1	2.2	11.6
CLIB 1284	0.3	**0.0**	11.3	1.6	1.6	7.6	4.4	1.1	2.5	6.0	1.0	11.6
NCYC 49	0.3	**0.0**	11.3	1.6	1.6	3.3	3.6	10.2	4.4	6.0	2.1	11.6
CLIB 1236	0.9	0.4	11.3	5.7	1.6	3.3	3.6	10.2	2.5	6.0	0.4	11.6
MUCL 881	1.5	0.3	11.3	5.7	1.6	3.3	3.6	10.2	2.5	**0.0**	-	11.6
FM 267	**0.2**	**0.2**, **0.1**	11.3	5.7	6.3	3.3	3.6	10.2	2.5	**0.0**	**0.1**	11.6
FM 119	3.7, 1.2	**0.1**	10.5	4.9, 0.6	5.5, 2.6	2.5, 6.8	3.6, 3.5	9.4	1.7, 3.6	2.8, 0	1.3	10.8
CBS 11176	4.6	**0.0**	11.5	5.8	6.4	3.4	4.5	10.4	**0.0**	6.2	4.0	11.7
CLIB 1267	4.6	**0.1**	11.5	5.8	6.4	3.4	4.5	1.2	1.4	6.2	4.0	11.7
CLIB 1235	0.4	**0.0**	11.5	5.8	6.4	3.4	4.5, 0	10.4	1.4	6.2	4.0	11.7
CNRZ 819	1.6	**0.1**	11.5	5.8	6.4	3.4	4.5	10.4	2.6	6.2	4.0	11.7
CBS 615.84	4.6, 1.6	0, 0.1	11.5	5.8	6.4, 1.7	3.4	4.5	10.4	1.4	6.2, **0.0**	4.0	11.7
CBS 182.33	1.0	**0.0**	11.5	5.8	6.4	3.4	4.4	10.4	2.6	6.2	4.0	11.7
CLIB 1248	1.0	**0.0**	11.5	5.8	6.4	3.4	4.5	10.4	**0.0**	6.2	4.0	11.7
CLIB 1283	0.6	1.0	11.5	5.8	6.4	3.4	4.5	10.4	4.5	**0.1**	4.0	11.7
FM 270	0.3	**0.0**	11.5	5.8	6.4	3.4	**0.0**	10.4	2.6	**0.1**	4.0	11.7
FM 125	4.6	**0.1**	11.5	0.3	6.4	3.4	4.5	10.4	1.4	6.2	4.0	**0.0**
FM 34	4.6	**0.1**	11.5	0.3	6.4	3.4	4.5	10.4	2.6	6.2	4.0	11.7

FM 268	4.6	**0.2**	11.5	0.3	6.4	3.4	4.5	10.4	2.6	**0.0**	4.0	11.7
CLIB 1274	**0.0**	**0.0**	11.5	5.8	6.4	3.4	4.5	10.4	1.4	3.7	0.6	11.7
FM 136	**0.0**	**0.3**	11.5	**0.5**	0.7	3.4	4.5	**0.0**	1.1	3.7	4.0	11.7

*) For each allele of each strain the presence or absence in the other 79 strains was quantified. Each correspondence between the allele of the index strain and that of one of the other strains was normalized with respect to the number of strains comprising the subject strain's clade as defined in figure 1, in order to account for over-, or under-sampling of the different clades. Since 13 groups are defined (9 large clades and 4 one- or two-strain groups), the maximum possible value is 13; the average value was 4.5. Values less than 5% of the average are highlighted in bold print. Strains are presented in the order of clades defined in figure 1.
