# Supplementary figures and images for: Molecular Genetic Analysis with Microsatellite-like Loci Reveals Specific Dairy-Associated and Environmental Populations of the Yeast Geotrichum candidum
Source: Microorganisms. 2022 Jan 4;10(1):103. doi: 10.3390/microorganisms10010103 (PMC8780849; doi:10.3390/microorganisms10010103)

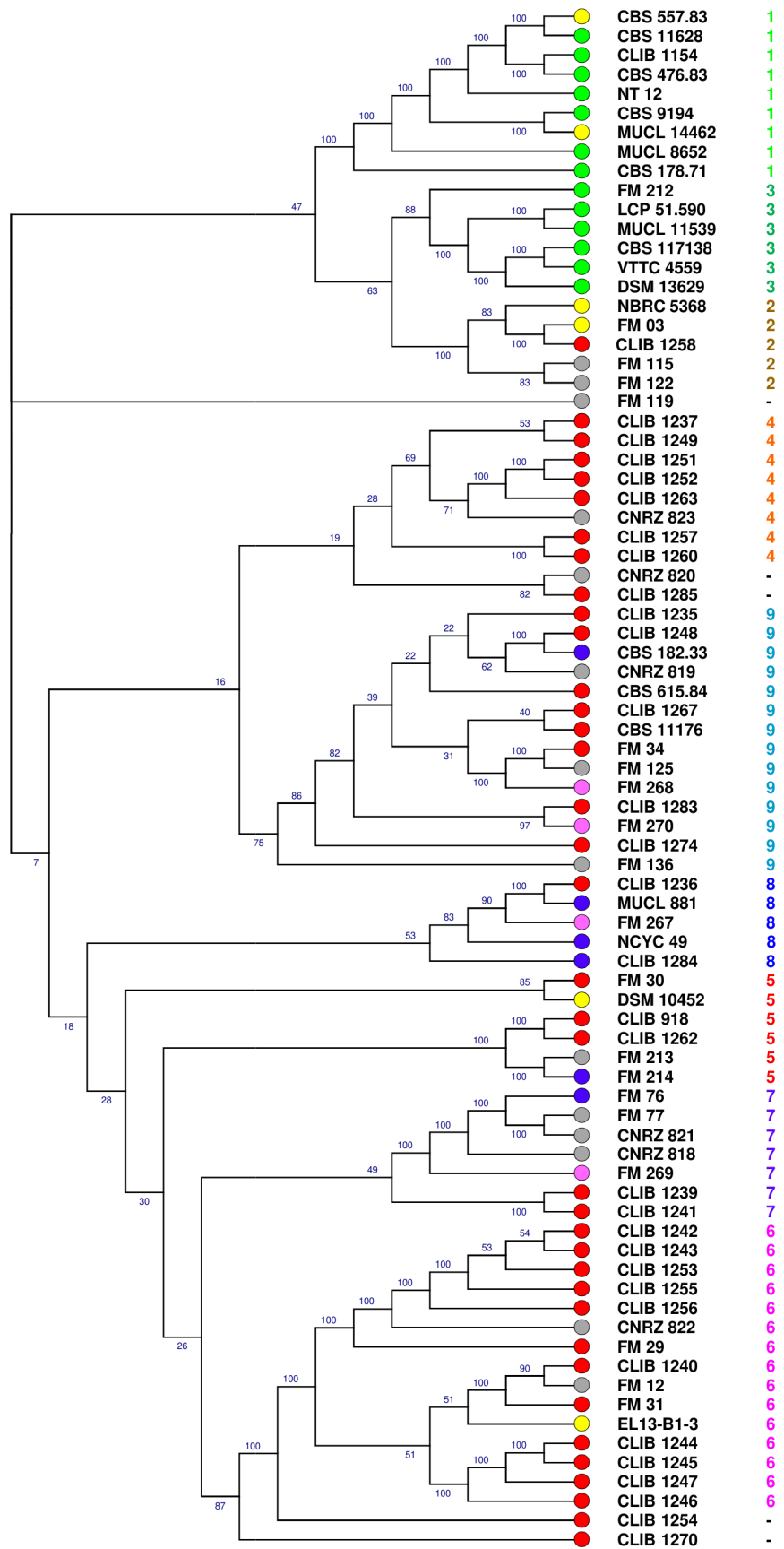

**Figure S2.** Phylogeny of the *G. candidum* strains, average of random haploid genotypes.

Supplement: Supplementary file 1 [file microorganisms-10-00103-s001.zip › Figure S2.pdf]
